# Soft tissue augmentation by autologous cultured fibroblasts transplantation for treatment of wrinkles and scars: a case series of 20 patients

**Published:** 2010

**Authors:** Mohammad Ali Nilforoushzadeh, Amir Hossein Siadat, Mojgan Arianrad, Fariba Moulavi, Elahe Haft Baradaran, Mohhamad Hossein Nasr Esfahani

**Affiliations:** a Dermatologist, Associate Professor of Dermatology, Head of Skin Diseases and Leishmaniasis Research Center (Sedigheh Tahereh), Tehran University of Medical Sciences, Tehran, Iran; b Dermatologist, Skin Diseases and Leishmaniasis Research Center (Sedigheh Tahereh), Isfahan University of Medical Sciences, Isfahan, Iran; c General Physician, Skin Diseases and Leishmaniasis Research Center (Sedigheh Tahereh), Isfahan University of Medical Sciences, Isfahan, Iran; d Rooyan Institute, Jahad Daneshgahi, Isfahan, Iran; e Embryologist, Rooyan Institute, Jahad Daneshgahi, Isfahan, Iran

**Keywords:** Soft Tissue Augmentation, Fibroblast, Wrinkle

## Abstract

**BACKGROUND:**

There are many filler agents for augmentation of static wrinkles and atrophic scars from synthetic, bio-synthetic, cadaver, animal and human sources.

**METHODS:**

The current study presents 20 patients whose facial wrinkles and lines were treated by transplantation of autologous cultured fibroblasts. The fibroblast nature of cells was confirmed by immune-staining and flow cytometry.

**RESULTS:**

The mean of improvement for this procedure at the 6 month follow up was 41%.

**CONCLUSIONS:**

In conclusion autologous fibroblast transplantation can be an effective procedure for correction of wrinkles and atrophic scars.

There are many filler agents for augmentation of static wrinkles and atrophic scars. These products are synthetic, bio-synthetic or derived from the cadaver, animal and human sources.^1^,^2^ However, the use of fillers has been associated with many side effects. Collagen, a major constituent of extra cellular matrix, derived from bovine source has been widely used as filler. It is reported that 6% of patients suffer hypersensitivity reactions to bovine collagen, which can manifest as granulomatous inflammation, necrosis, or abscess formation. Rare systemic complications have also been reported.^3^

The ideal filler would be an autologous, injectable material that provides long-term results, requires minimal surgery and tissue removal for initial tissue harvest, and has unlimited yield without the need for additional tissue harvest. Some disadvantages of synthetic or biosynthetic fillers include the possibility of allergy and short duration of their effect. Therefore, recent efforts are focused on trying to use autologous approaches like injecting the autologous collagen and autologous cultured fibroblasts transplantation.^4^,^5^ The latter procedure satisfies the criteria for suitable filler. Therefore, in the current study, 20 patients were presented whose facial wrinkles and lines were treated by injecting of autologous cultured fibroblasts with 6 month follow ups.

## Methods

Autologous transplantation of fibroblast has achieved FDA approval. However, this study was also initially approved by the ethical committee of Isfahan University of medical science.

### 

#### Skin Biopsy

Initially, a 4 mm^2^ punch biopsy was taken from the retro-auricular region. This site is thought to be ideal since it is not exposed to sun, is well vascularised region and is suitable for creating a small scar. The biopsy site was anesthetized using field block technique. Then, 50 cc blood was taken from the patients and both the skin sample at 4°C and the blood were transferred to Rooyan Research Center for fibroblast isolation and culture.

#### Fibroblast Isolation and Culture

Initially, the fatty tissue was removed and the skin samples were minced into small pieces and placed onto culture dishes containing DMEM F12 + 10% AHS (autologous human serum) and 100 μg/ml streptomycin, 100 IU/ml penicillin and 2 mM L-Glutamine at 37°C and 5% CO2 and 95% humidity. Upon culture, the fibroblasts expanded out of the explants and reach confluency. Finally, 40 million cells were isolated from each biopsy and prepared for transplantation. Cells viability was assessed by trypan blue staining.

#### Autologous Fibroblast Transplantation

EMLA cream (Company) was applied 1 hour before transplantation and 20 million cells were implanted in dermal and sub dermal tissue. All the patients received cell transplantation in the nasolabial fold. Injections were repeated 2 times, 2 weeks apart.

#### Physician Improvement Rating

**Figure 1:**
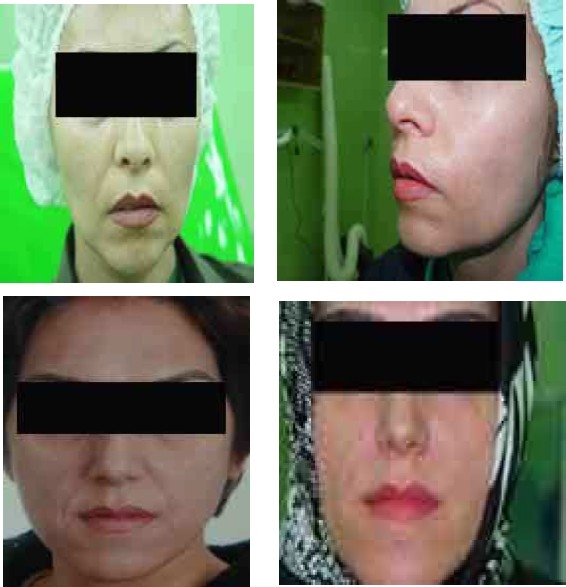
Photographs of 2 different patients before and 6 month after treatment with autologous fibroblasts

Improvement was assessed by two expert physicians that were not involved in this study. All patients were examined before and 6 month after the treatment. Captured images were used to aid physician assessments (Figure 1).

#### Cell Specific Markers

Phenotype of fibroblasts was confirmed using immunocytochemical staining by monoclonal antibodies directed against the vimentin. In summary, growth of cells was continued to confluency in 24 well dishes over glass cover slips. After washing the cell with PBS, they were fixed in methanol at 4°C for 20 minutes. The cells were then washed in PBS. PBS was composed of 139 mM NaCl, 2.7 mM KCl, 4.3 mM Na2HPO4, 7H2O, 1.48 mM KH2PO4. After washing, they were blocked with 3% BSA in PBS for 15 minutes at 37°C. The block was then removed and 100 μl of 1:40 dilution antivimentin clone V9 (Sigma, catalog no. V- 6630) was added. Incubation of slides was performed for 1 hour at 37°C. After washing the cells with PBS, they were incubated with 100 ml of a 1:300 dilution of FITC-labeled anti-mouse IgG for one hour. Cells were then washed with PBS and counter stained with Propidium iodide in PBS for 10 seconds.

Finally, the cells were washed with PBS and covered with 50% glycerol and were observed by fluorescence microscopy. Proper autofluorescence and secondary antibodies controls were included.

In order to evaluate the percentage of cells which express fibroblast cell specific markers, the ethanol permeableized cells were centrifuged (10 minutes, 3000 rpm) and resuspended in PBS. The suspension was syringed through a 30 G needle before staining. Cell suspension was blocked by 10% normal goat serum (Chemicon S26) for 45 minutes. The blocked were removed and cells were treated with 1:100 anti-vimentin dilution and isotype controls which included purified mouse IgG (Chemicon CBL600). Goat anti-mouse FITC (Chemicon AP124F) were used as secondary antibodies at 1:50 dilution. Cells were analyzed within 24 hours by flow cytometery (BD, FACs Calibur).

## Results

Out of 20 individuals participating in this study, 17 patients (85%) were female and 3 patients (15%) were male with the minimum and maximum age of 40 and 62, respectively. All the 20 individuals received autologous fibroblast in the nasolabial fold. The mean improvement for autologous fibroblast transplantation at 6 month follow up was 41 ± 13% with minimum of 20% and maximum of 60%. There was no report of side effects or allergic reaction, except small reddening around the treatment area which disappeared within 24 hours of cell transplantation.

## Discussion

It is stated that autologous transplantation of skin fibroblast may be a safe product for sustained improvements in contour defects without surgery and virtually zero risk of hypersensitivity reactions.^6^

Histological analysis in different studies have shown that fibroblast injections increase collagen formation along with increase in thickness and density of dermal collagen without induction of an inflammatory response.^6^–^8^

In the current study, the efficacy of fibroblast injection for treatment of nasolabial fold was evaluated. The results show that fibroblast injection is useful for improvement of nasolabial fold and possibly for scars and wrinkles. Although the degree of improvement is variable between individuals, no significant side effect was observed in the present patients.

To our best knowledge, it is the first experience of using autologous fibroblast provided in Asia.

In a study performed by Weiss et al,^6^ the efficacy and side effects of autologous living fibroblast injections versus placebo in a randomized phase III trial were evaluated for the treatment of various facial contour defects. The results showed that living fibroblasts produced significantly greater improvements in dermal deformities and acne scars than did placebo. At 9- and 12-month follow-ups, live fibroblast-treated patients continued to demonstrate benefit from treatment with response rates of 75.0% and 81.6%, respectively. Similar to the present study no serious treatment-related adverse events were reported.^6^

In another study, the effectiveness of intradermal injections of autologous fibroblasts for the treatment of facial rhytids and dermal depressions was performed in ten adults (age range of 24-69 years) each of who exhibited a prominent rhytid or depressed facial scar. Nine of 10 patients noted a 60% to 100% improvement with the treatment; clinicians made similar observations. Size reduction of 10% up to 85% of the study site was demonstrated by optical profilometry for every patient. Microscopically, there was evidence of increased thickness and density of dermal-layer collagen.^7^ The different improvement rate between patients can be due to the age variation, proper injection of cell in the right skin level, differences in the degree of nasolabial fold, number of injection and duration of assessment. Hyaluronic acid belongs to a family of macromolecules known as glycosaminoglycans. These molecules, composed of chains of repeating disaccharide units, are extensively found in the native extracellular matrix of connective tissues. The hydrophilic composition of glycosaminoglycans attracts water into the extracellular matrix conferring a degree of turgor to the tissue. After transplantation, the hyaluronic acid derivatives undergo local degradation. The metabolic products are then further catabolized by the liver into carbon dioxide and water. Commercially available injectables are gels of hyaluronic acid derivatives that have been cross-linked to prolong their degradation in vivo. Two large series of patients have confirmed the duration of the augmentation achieved with hyaluronic acid derivatives to be 9 months.^9^

However, regarding fibroblast injection, recent studies have demonstrated objectively and subjectively measured improvements in facial contour defects lasting at least 12 to 48 months.^6^ Therefore, although the injection of hyaluronic acid derivatives seems to be cheaper at first glance, the need for repeated injection for hyaluronic acid derivatives makes the cost of these 2 treatment methods almost equal. On the other hand, there is still a need to perform a small punch biopsy for obtaining tissue needed to perform culture.

The results of immnocytochemical staining and flow cytometery revealed that the recovered cells have fibroblast identity and over 91.84% of cells were antivimentin positive. The trypan blue staining reveals that over 95% of cells were viable before transplantation.

## Conclusions

In conclusion fibroblast injection can be an effective method for correction of wrinkles and atrophic scars. This method seems to have low side effects although studies with longer follow up seem to be mandatory.
